# Longitudinal relationships of depressive symptoms and cognition in older adults grouped by depression age of onset

**DOI:** 10.1093/geroni/igaf122.3231

**Published:** 2025-12-31

**Authors:** Noah Green, Monica Walters, Laura Zahodne

**Affiliations:** University of Michigan, Ann Arbor, Michigan, United States; University of Michigan, Ann Arbor, Michigan, United States; University of Michigan, Ann Arbor, Michigan, United States

## Abstract

Depression is a known risk factor for cognitive impairment in older adults; however, it is unclear how age of onset moderates concurrent and longitudinal relationships between depressive symptoms and cognition in later life. This longitudinal study examines the relationship between depressive symptoms and episodic memory in older adults reporting a history of depression across different life stages. Participants included 373 adults aged 55+ from the Michigan Cognitive Aging Project (MCAP), who reported at least one lifetime depressive episode. Episodic memory was operationalized as the sum of learning trials from the CERAD word list learning task. Depressive symptoms were measured using the ten-item Center for Epidemiological Studies Depression Scale. Age of onset was categorized into four groups according to first depressive episode (childhood [ages < 12; n = 69], adolescence [ages 13-17; n = 73], early adulthood [ages 18-44; n = 155], middle adulthood [ages 45-59; n = 55], late adulthood [ages 60+; n = 21]. Cross-lag panel analyses stratified by age of onset modeled relationships between depressive symptoms and episodic memory across three time points over four years, while controlling for age, gender, race, and education. Longitudinal associations between more depressive symptoms and worse subsequent memory were only identified among adolescent (β=-0.241, p = 0.017) and early adulthood (β=-0.139, p = 0.026) onset groups. A longitudinal association between worse memory and more subsequent depressive symptoms was only identified among those with early adulthood onset (β=-0.270, p = 0.010). Results point to potential critical developmental periods for depression onset with implications for later life prognosis and/or mechanisms involving depressive symptoms and cognitive aging.

